# AIPSS‐MF machine learning prognostic score validation in a cohort of myelofibrosis patients treated with ruxolitinib

**DOI:** 10.1002/cnr2.1881

**Published:** 2023-08-08

**Authors:** Andrea Duminuco, Adrian Mosquera‐Orgueira, Antonella Nardo, Francesco Di Raimondo, Giuseppe Alberto Palumbo

**Affiliations:** ^1^ Hematology with BMT Unit, A.O.U. “G. Rodolico‐San Marco” Catania Italy; ^2^ Department of Haematology Guy's and St Thomas NHS Foundation Trust London UK; ^3^ Hospital Clínico Universitario Santiago de Compostela Spain; ^4^ Dipartimento di Specialità Medico‐Chirurgiche, CHIRMED University of Catania Catania Italy; ^5^ Dipartimento di Scienze Mediche Chirurgiche e Tecnologie Avanzate “G.F. Ingrassia” University of Catania Catania Italy

**Keywords:** AIPSS‐MF, machine learning, myelofibrosis, RR6, ruxolitinib, standard prognostic score

## Abstract

**Background:**

In myelofibrosis (MF), new model scores are continuously proposed to improve the ability to better identify patients with the worst outcomes. In this context, the Artificial Intelligence Prognostic Scoring System for Myelofibrosis (AIPSS‐MF), and the Response to Ruxolitinib after 6 months (RR6) during the ruxolitinib (RUX) treatment, could play a pivotal role in stratifying these patients.

**Aims:**

We aimed to validate AIPSS‐MF in patients with MF who started RUX treatment, compared to the standard prognostic scores at the diagnosis and the RR6 scores after 6 months of treatment.

**Methods and results:**

At diagnosis, the AIPSS‐MF performs better than the widely used IPSS for primary myelofibrosis (C‐index 0.636 vs. 0.596) and MYSEC‐PM for secondary (C‐index 0.616 vs. 0.593). During RUX treatment, we confirmed the leading role of RR6 in predicting an inadequate response by these patients to JAKi therapy compared to AIPSS‐MF (0.682 vs. 0.571).

**Conclusion:**

The new AIPSS‐MF prognostic score confirms that it can adequately stratify this subgroup of patients already at diagnosis better than standard models, laying the foundations for new prognostic models developed tailored to the patient based on artificial intelligence.

## 1 INTRODUCTION

Myeloproliferative neoplasms (MPNs) are a group of hematological disorders distinguished by heterogeneity in presentation and variable clinical course. Among them, myelofibrosis (MF) is the condition with the worst life expectancy due to several underlying clinical, pathological, and molecular factors. This condition, divided into primary (PMF) or secondary (SMF), occurring after another MPN, is characterized by variable overall survival (OS), with a range from <2 to 20 years.^1^ Several prognostic factors evaluated both at the time of diagnosis and later during the follow‐up of the disease are extensively considered in a few currently available prognostic scores, that is, International Prognostic Score System (IPSS), Dynamic IPSS (DIPSS) for PMF, and Myelofibrosis Secondary to PV and ET‐Prognostic Model (MYSEC‐PM) for SMF.^2^ The more accurate prognostic models (Genetically Inspired Prognostic Scoring System (GIPSS), Mutation‐Enhanced International Prognostic Score System for Transplantation‐Age Patients (MIPSS70) and v.2) consider the molecular and genomic side, through the wide use of the next‐generation (NGS) able to identify the occurrence of high‐risk mutations.^3^

Beyond these, several working groups propose new scores to improve the ability to better identify patients with the worst outcome. In this context, the AIPSS‐MF (Artificial Intelligence Prognostic Scoring System for Myelofibrosis, based on machine learning) could play a pivotal role as it is based exclusively on clinical and easily accessible variables at diagnosis and has been shown to outperform the prognostic accuracy of the IPSS in PMF and the MYSEC‐PM in SMF.^4^ Over the years, various drugs have been authorized or are ongoing tested for treating these conditions.^5^ The finding of driver mutations such as JAK2, MPL, and CALR has changed the understanding and management of MF.^3^ Consequently, the availability of JAK inhibitor (JAKi) drugs, first of all, ruxolitinib, has revolutionized the treatment approach of MF.^6^ This drug can ensure rapid reduction of symptoms and spleen size while leading to quality of life improvement in most patients and allowing younger and fit patients to undergo allogeneic bone marrow transplantation (HSCT), the only curative therapy currently available, in a better condition. Recently, in the setting of patients undergoing ruxolitinib therapy, a prognostic model named Response to Ruxolitinib after 6 months (RR6) for stratifying these patients in risk classes based was set, identifying “early” predictors (after 6 months of ruxolitinib) of inferior survival: the ruxolitinib administered dose, the palpable spleen length reduction, and the red blood cells transfusion requirement.^7^ Furthermore, the effectiveness of this model has been recently externally validated by different working groups,^8^, ^9^ moreover being able to identify patients who discontinue treatment early due to poor response or toxicities (hematological or not).^10^, ^11^, ^12^

The role of machine learning quickly plays a central role in medicine, and data extrapolated from real‐life experience could strengthen the impact and large‐scale applicability, also in the hematology field, where it could be helpful in narrowing a differential diagnosis, aiding therapy selection, generating risk prediction models, helping the physician in avoid medical errors, thus improving productivity.^13^ A validation of the performance of AIPSS‐MF in a cohort of patients treated with ruxolitinib can confirm the role of machine learning to ensure a more suitable prognostic stratification than the common ones also in this setting of patients.

## 2 METHODOLOGY

### 2.1 Aims of the study and cohort's features

This retrospective observational report aimed to validate the artificial intelligence model AIPSS‐MF in patients with MF who started ruxolitinib treatment during the follow‐up, comparing it to the standard prognostic scores calculated at the diagnosis (IPSS and MYSEC‐PM). The second endpoint was to evaluate the same model when the ruxolitinib started, comparing it with the RR6 prognostic score assumed as the gold standard, and determine if the AIPSS‐MF model score calculated at the diagnosis can perform adequately also this setting of patients. Our cohort was based on 103 adult (>18 years) patients affected by MF, ineligible to HSCT (due to patients' choice, comorbidities, and availability of potential donor), and referred to the Hematology Unit with Bone Marrow Transplantation, Policlinico “G. Rodolico” – San Marco, Catania, Italy, and treated with ruxolitinib as the first line of treatment. Exclusion criteria were a life expectancy of fewer than 6 months, ECOG >2, and previous allogeneic HSCT. Fifty seven patients (55.3%) were affected by PMF and 46 (44.7%) by SMF. Patient demographic data and laboratory parameters were recorded at diagnosis, just before ruxolitinib was started, and after 3 and 6 months of treatment. The data are shown in Table 1. According to the guidelines, the ruxolitinib dose at the start of treatment and during follow‐up was chosen based on platelet value. The median time to treatment from MF diagnosis to initiation of ruxolitinib therapy was 8.1 months (IQR 2.4–25.5). At the end of the observation period (from December 2002 to November 2022), 42 patients (40.8%) were still on ruxolitinib treatment. Of 61 patients who discontinued treatment, 27 died of MF progression, 7 transformed into acute myeloid leukemia, 5 of respiratory failure (of which 2 SARS‐CoV‐2 infections due to impaired vaccine's immune response^14^), 8 of different neoplasms (of which 3 from non‐melanoma skin cancer likely ruxolitinib‐related), 8 of clotting/bleeding event (e.g., myocardial ischemia, cerebral hemorrhage), the other from MF/treatment‐unrelated causes. Twenty patients interrupted ruxolitinib due to intolerance/refractory disease. Median overall survival (mOS) (defined as the time from MF diagnosis to death from any cause) was 95.04 months (reported in Figure S1).

**TABLE 1:** Features of enrolled patients at diagnosis and at ruxolitinib treatment start.

	At diagnosis	At RUX treatment start
Median age, years (range)	68.4 (38–84)	69.4 (38–83)
Sex M/F, *n* (%)	62 (60.2)/41 (39.8)	/
PMF, *n* (%)	57 (55.4)	/
SMF, *n* (%)	46 (44.7)	/
PET‐MF, *n* (%)	22 (21.4)	/
PPV‐MF, *n* (%)	24 (23.3)	/
BM fibrosis grade 0/1/2/3, *n* (%)	1 (0.9)/39 (37.9)/46 (44.7)/17 (16.5)	
Mutation status		
JAK2‐mutated, *n* (%)	80 (77.7)	/
CALR‐mutated, *n* (%)	15 (14.6)	/
MPL‐mutated, *n* (%)	3 (1.9)	/
“Triple negative”, *n* (%)	6 (5.8)	/
Normal/abnormal karyotype, *n* (%)	98 (95.2)/5 (4.8)	/
PMF: IPSS/DIPSS^a^ Low/Int‐1/Int‐2/HR, *n* (%)	8 (14.0)/19 (33.3)/22 (38.7)/8 (14.0)	0 (0)/15 (26.3)/32 (56.2)/10 (17.5)
SMF: MYSEC‐PM Low/Int‐1/Int‐2/HR, *n* (%)	3 (6.5)/16 (34.8)/20 (43.5)/7 (15.2)	0 (0)/16 (34.8)/19 (41.3)/11 (23.9)
Median WBC, ×10^9^/L (IQR)	10.0 (7.1–17.0)	12.3 (7.0–19.6)
Median Hb (g/dL) (IQR)	10.8 (8.6–13)	10.0 (8.8–12.0)
Median PLT × 10^9^/L (IQR)	372 (222–562)	308 (174–498)
Presence of 1%–3% blasts in PB, *n* (%)	4 (3.9)	7 (6.8)
Constitutional symptoms Y/N, *n* (%)	61 (59.2)/42 (40.8)	87 (84.5)/16 (15.5)
RBC 3 months before RUX Y/N—*n* (%)	/	29 (28.1)/74 (71.9)
RUX dose < 20 mg twice a day—*n* (%)	/	38 (18.9)

Abbreviations: BM, bone marrow; Hb, hemoglobin; IQR, interquartile range; PB, peripheral blood; PLT, platelets; RBC, red blood cells support; RUX, ruxolitinib; WBC, white blood cells.

^a^ IPSS score used at diagnosis, DIPSS at treatment start.

## 3 STATISTICAL ANALYSIS

The discriminative capacity of the models was evaluated with out‐of‐bag estimates of the concordance index (C‐index). The precision of the AIPSS‐MF score compared to the other prognostic model was assessed using cross‐validated time‐dependent areas under the curve (AUCs) and evaluated in four different time points (2.5, 5, 7.5, and 10 years) derived from the Cox survival models. A comparison of data collected at the time of diagnosis and at the time of the start of ruxolitinib therapy was made. Statistical analysis was performed using R‐commander software (R Foundation for Statistical Computing, Vienna, Austria. https://www.R-project.org/).

## 4 RESULTS

**FIGURE 1:**
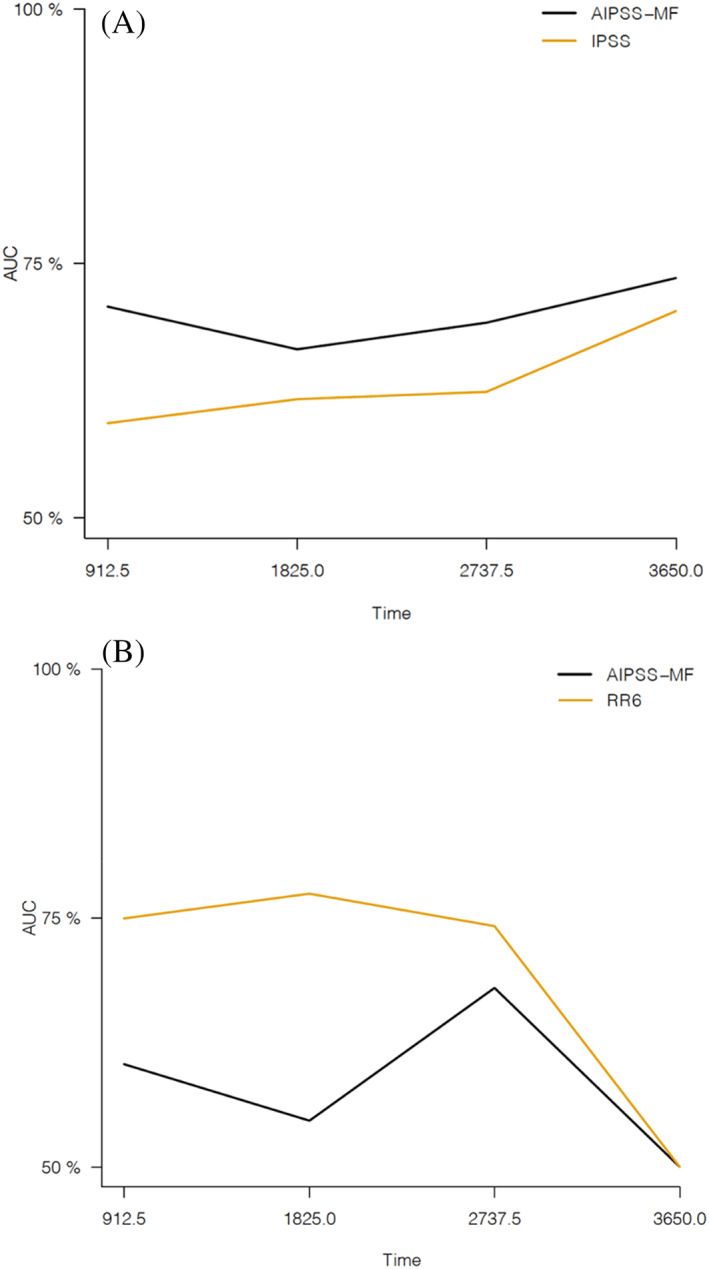
Evidence of AIPSS‐MF performance correlated with IPSS at diagnosis (A) and with RR6 based on data extrapolated 6 months after ruxolitinib therapy (B). The time in abscissa is reported in days. AIPSS‐MF, artificial intelligence prognostic scoring system for myelofibrosis; IPSS, international prognostic score system; RR6, response to ruxolitinib after 6 months.

At diagnosis, in the whole cohort, the AIPSS‐MF performs better than the widely used and recognized IPSS (Figure 1A). Then, splitting patients into PMF and SMF, we compared the new score with IPSS for PMF and MYSEC‐PM for SMF, despite the reduced statistical significance for the small size of the samples of patients (57 and 46 patients, respectively). In these cases, the AIPSS‐MF model confirms its superiority versus IPSS for patients with PMF (C‐index 0.636 vs. 0.596). In the SMF setting, adequate patient stratification is not possible due to the small sample size because, during bootstrapping, we reported a model failure. However, the AIPSS‐MF model, compared to MYSEC‐PM, maintains a better ability to predict OS at diagnosis (C‐index 0.616 vs. 0.593). Analysis data with standard errors are reported in Table S1.

On the other hand, the analyses performed with data extrapolated at the start and during the first 6 months of ruxolitinib therapy confirmed the leading role of RR6 in early predicting an inadequate response by these patients to JAKi therapy. The RR6 model achieved a higher AUC at all evaluated times points compared with the AIPSS‐MF, reaching a superimposable rate for both models at 10 years (Figure 1B). The 2.5, 5, and 7.5‐year AUCs of the RR6 model were 75.8%, 77.9%, and 73.7%, compared to 62.3%, 54.4%, and 67.6% of the artificial intelligence model. The C‐index confirmed the superiority of RR6 (0.682 vs. 0.571). The same results are achieved by subdividing the patients in PMF and SMF (C‐index 0.674 and 0.679 for RR6 vs. 0.561 and 0.599 for AIPSS‐MF, respectively). The whole analysis data with standard errors are reported in Table S2.

## 5 DISCUSSION

MIPSS70, v.2, and GIPSS are certainly the prognostic models able to better stratify patients with MF. These scores, however, required cytogenetic and molecular data, often not easily accessible, above all in small centers where more advanced risk stratification techniques (NGS) are not readily available, or in case of failure to obtain bone marrow samples (for example dry tap, frequent in fibrotic marrow). The new AIPSS‐MF prognostic score, using clinical variables could represent a turning point in these events.

Despite the limitations in terms of the size of groups (especially for SMF, where the small number of patients led to a failure in bootstrapping), and the no consideration of molecular and genetics variables (that remain the milestone in the building of prognostic models), the AIPSS‐MF prognostic score can be used when diagnosing myelofibrosis in patients with PMF and SMF, especially in small centers, also in a selected cohort of patients who requested active treatment for MF, with better results than standard model scores (IPSS and MYSEC‐PM). To perform our analysis, we tried to reduce the bias factors that could lead to a broad heterogeneity of the cohort excluding patients who experienced previously or were subsequently addressed to HSCT, including only patients in first‐line treatment for MF.

On the other side, RR6 remains the better useful prognostic model with clear superiority in shorter follow‐ups, being precisely built with data collected after drug initiation to identify poor responders to ruxolitinib that could benefit a treatment shift. Larger multicentric cohorts with the exclusion of confounding factors or bias are necessary to confirm these findings.

The development of individualized prognostic models represents the future.^15^ Patient's specific characteristics (disease's features,^15^ concurrent comorbidities^16^) pave the way to personalized prognosis, ensuring the best management. Machine learning represents a visionary approach to identifying crucial information to frame the patient and the best treatment plan, promising improvements in sight with shocking speed.^17^ Thus, starting from these assumptions and confirming them in larger multicentric patient databases, a new model for JAKi response could be created by exploiting the great potential of machine learning in medicine.

## 6 CONCLUSION

AIPSS‐MF remains a useful and easily accessible prognostic model for the stratification of MF also in ruxolitinib‐treated patients, despite the RR6 remains the gold standard model to early identify patients who could benefit from a treatment‐shift.

## AUTHOR CONTRIBUTIONS

**Andrea Duminuco:** Conceptualization (equal); data curation (lead); formal analysis (equal); writing – original draft (lead); writing – review and editing (equal). **Adrian Mosquera‐Orgueira:** Formal analysis (equal). **Antonella Nardo:** Data curation (equal). **Francesco Di Raimondo:** Supervision (equal); writing – review and editing (equal). **Giuseppe Alberto Palumbo:** Conceptualization (equal); supervision (equal); writing – review and editing (equal).

## CONFLICT OF INTEREST STATEMENT

Giuseppe Alberto Palumbo received honoraria from Abbvie, AOP, AstraZeneca, BMS Celgene, GSK, Incyte, Janssen, MorphoSys, Novartis. Andrea Duminuco received honoraria from BMS Celgene, Incyte, EusaPharma. Francesco Di Raimondo received honoraria from Amgen, Janssen, Celgene, Takeda. All the other authors declare no conflict of interest.

## ETHICS STATEMENT

The study was conducted in accordance with the Declaration of Helsinki and approved by Ethical Committee Catania‐1. Informed consent was obtained from all subjects involved in the study. No funding to declare.

## Supporting information


**FIGURE S1.** Overall Survival expressed in months for 103 enrolled patients affected by primary or secondary myelofibrosis. mOS = 95.04 months.
**TABLE S1.** AUC analysis in the context of primary and secondary MF, evaluated at diagnosis. AUC: area under the curve.
**TABLE S2.** AUC analysis in the context of primary and secondary MF, evaluated at the start of ruxolitinib's treatment. AUC: area under the curve.

## Data Availability

The data that support the findings of this study are available from the corresponding author upon reasonable request.
