# Supplementary material for: AIPSS‐MF machine learning prognostic score validation in a cohort of myelofibrosis patients treated with ruxolitinib
Source: Cancer Rep (Hoboken). 2023 Aug 8;6(10):e1881. doi: 10.1002/cnr2.1881 (PMC10598243; doi:10.1002/cnr2.1881)
Supplement: Supplementary file 1 — FIGURE S1. Overall Survival expressed in months for 103 enrolled patients affected by primary or secondary myelofibrosis. mOS = 95.04 months. TABLE S1. AUC analysis in the context of primary and secondary MF, evaluated at diagnosis. AUC: area under the curve. TABLE S2. AUC analysis in the context of primary and secondary MF, evaluated at the start of ruxolitinib's treatment. AUC: area under the curve. [file CNR2-6-e1881-s001.docx]

# Supplemental data

*Supplementary Figure 1. Overall Survival expressed in months for 103 enrolled patients affected by primary or secondary myelofibrosis. mOS = 95.04 months*


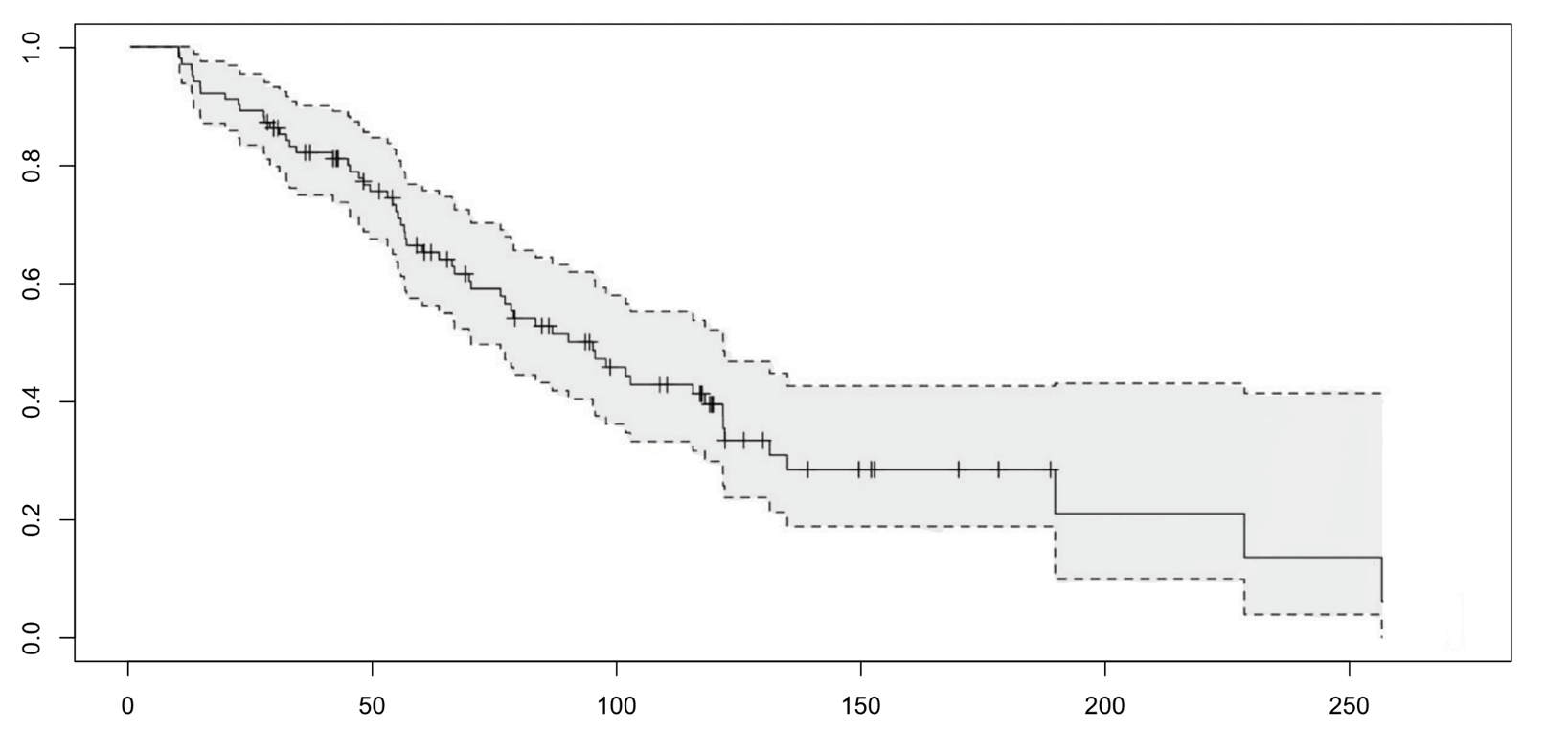


*Supplementary Table 1. AUC analysis in the context of primary and secondary MF, evaluated at diagnosis. AUC: area under the curve*

| All patients | | | | | |
| --- | --- | --- | --- | --- | --- |
| Time-points | | 2.5 y | 5 y | 7.5 y | 10 y |
| AIPSS-MF | AUC | 70.76 | 66.56 | 69.18 | 73.56 |
| IPSS |  | 59.30 | 61.66 | 69.18 | 73.56 |
|  |  |  |  |  |  |
| *Coxph model* |  | c-index | Standard error | | |
| AIPSS-MF |  | 0.643 | 0.04 |  |  |
| IPSS |  | 0.609 | 0.04 |  |  |
|  |  |  |  |  |  |
|  |  |  |  |  |  |
| Primary MF IPSS-evaluated patients (N=57) | | | | | |
|  |  |  |  |  |  |
| *Coxph model* |  | c-index | Standard error | |  |
| AIPSS-MF |  | 0.636 | 0.05 |  |  |
| IPSS |  | 0.596 | 0.05 |  |  |
|  |  |  |  |  |  |
|  |  |  |  |  |  |
| Secondary MF MYSEC-PM-evaluated patients (N=46) | | | | | |
|  |  |  |  |  |  |
| *Coxph model* |  | c-index | Standard error | |  |
| AIPSS-MF |  | 0.616 | 0.07 |  |  |
| MYSEC-PM |  | 0.593 | 0.06 |  |  |

*Supplementary Table 2. AUC analysis in the context of primary and secondary MF, evaluated at the start of ruxolitinib’s treatment. AUC: area under the curve*

| All patients | | | | | |
| --- | --- | --- | --- | --- | --- |
| Time-points | | 2.5 y | 5 y | 7.5 y | 10 y |
| AIPSS-MF | AUC | 62.25 | 54.39 | 67.61 | 50.00 |
| RR6 |  | 75.78 | 77.89 | 73.74 | 50.00 |
|  |  |  |  |  |  |
| *Coxph model* |  | c-index | Standard error | | |
| AIPSS-MF |  | 0.571 | 0.04 |  |  |
| RR6 |  | 0.682 | 0.03 |  |  |
|  |  |  |  |  |  |
|  |  |  |  |  |  |
| Primary MF IPSS-evaluated patients (N=57) | | | | | |
|  |  |  |  |  |  |
| *Coxph model* |  | c-index | Standard error | |  |
| AIPSS-MF |  | 0.561 | 0.04 |  |  |
| RR6 |  | 0.674 | 0.04 |  |  |
|  |  |  |  |  |  |
|  |  |  |  |  |  |
| Secondary MF MYSEC-PM-evaluated patients (N=46) | | | | | |
|  |  |  |  |  |  |
| *Coxph model* |  | c-index | Standard error | |  |
| AIPSS-MF |  | 0.599 | 0.06 |  |  |
| RR6 |  | 0.679 | 0.05 |  |  |
